# Psychological first aid for workers in care and nursing homes: systematic review

**DOI:** 10.1186/s12912-022-00866-6

**Published:** 2022-04-26

**Authors:** Mariyana Schoultz, Claire McGrogan, Michelle Beattie, Leah Macaden, Clare Carolan, Rob Polson, Geoffrey Dickens

**Affiliations:** 1 Faculty of Health and Life Sciences, Northumbria University, Newcastle, England; 2 Centre for Health Sciences, University of the Highlands and Islands, Inverness, Scotland; 3 University of the Highlands and Islands, Stornoway, Scotland

**Keywords:** Psychological first aid, PFA, Care homes, Nursing homes, Systematic review, COVID-19, Care staff

## Abstract

**Background:**

The Covid-19 pandemic has produced unprecedented challenges across all aspects of health and social care sectors globally. Nurses and healthcare workers in care homes have been particularly impacted due to rapid and dramatic changes to their job roles, workloads, and working environments, and residents’ multimorbidity. Developed by the World Health Organisation, Psychological First Aid (PFA) is a brief training course delivering social, emotional, supportive, and pragmatic support that can reduce the initial distress after disaster and foster future adaptive functioning.

**Objectives:**

This review aimed to synthesise findings from studies exploring the usefulness of PFA for the well-being of nursing and residential care home staff.

**Methods:**

A systematic search was conducted across 15 databases (Social Care Online, Kings Fund Library, Prospero, Dynamed, BMJ Best Practice, SIGN, NICE, Ovid, Proquest, Campbell Library, Clinical Trials, Web of Knowledge, Scopus, Ebsco CINAHL, and Cochrane Library), identifying peer-reviewed articles published in English language from database inception to 20th June 2021.

**Results:**

Of the 1,159 articles screened, 1,146 were excluded at title and abstract; the remaining 13 articles were screened at full text, all of which were then excluded.

**Conclusion:**

This review highlights that empirical evidence of the impact of PFA on the well-being of nursing and residential care home staff is absent. PFA has likely been recommended to healthcare staff during the Covid-19 pandemic. The lack of evidence found here reinforces the urgent need to conduct studies which evaluates the outcomes of PFA particularly in the care home staff population.

## Introduction

The COVID 19 pandemic has produced unprecedented challenges across all aspects of health and social care sectors globally. Nurses and healthcare workers (HCWs) in care homes have been particularly impacted due to rapid and dramatic changes to their job roles, workloads, working environments, and their residents’ mortality and multimorbidity [1, 2].

There are approximately half a million people living in nursing and residential care homes across the UK and almost 1.8 million staff working in these settings [3]. During the early stages of the pandemic, government resources were directed towards acute care contexts and support for care homes was slow in arriving and limited in scope, despite an urgent need [4]. The slow response likely impacted on the high mortality of residents in these settings. Twenty one percent of respondents to a UK-wide survey [5] reported that their care home accepted COVID 19 positive patients being discharged from hospital, and a further 43% said their care home accepted admissions of patients whose COVID 19 status was unknown, while only two thirds of participants said they always had access to appropriate personal protective equipment. Since the start of the pandemic, 24% of deaths in UK care homes involved COVID 19, with 35,000 excess deaths reported in the first two months, compared to the average number for this period. During both the first and second waves, up to 72% of deaths involving COVID 19 worldwide were care home residents [1] and COVID 19 was implicated in more deaths of male care home residents than any other cause [6]. Given that HCWs often form close and long-term working relationships with residents, it is unsurprising that studies have reported significant negative mental health impacts on HCWs during the pandemic. A systematic review [7] of well-being among HCWs during the pandemic found a high prevalence of anxiety (23.3%), depression (22.8%), and insomnia (38.8%). Similarly, a Queen’s Nursing Institute report [5] and rapid review by Embregts et al. [8] and Kisely et al. [9] identified that staff working in care homes during the pandemic experienced a range of negative emotional and psychological effects such as fear, stress, tension, as well as experiences of moral injury, perceived lack of support, and blame for residents’ deaths. In a separate study, 20% of survey respondents stated they had considered leaving the care profession due to the impact of COVID 19 on their working conditions [10]. It is important to note that the additional pressures and uncertainties associated with the pandemic possibly exacerbated the challenges faced by care home staff pre-pandemic [11]. For example, it has been found that HCWs are likely to experience post-traumatic stress disorder (PTSD) symptoms due to many unpredictable occupational stresses (acute and chronic) such as sudden death, dealing with residents with psychological trauma or violent behaviour [12, 13]. Further, evidence suggests that there is a link between care home workers with PTSD and poor quality of care for residents [14]. Taken together, these findings highlight a clear and urgent need for effective well-being interventions for staff working in these settings, both in the current context of the pandemic and beyond.

One such intervention designed to alleviate the impact of crisis on mental health and well-being is Psychological First Aid (PFA). Developed by the World Health Organisation [15], PFA is a brief training course that aims to reduce initial distress, meet current needs (psychological and physical), promote flexible coping, and encourage adjustment while establishing feelings of safety, calmness, self- and community efficacy, connectedness, and hope. The PFA does not include discussions about the traumatic events, but focuses on providing practical care and support, by assessing the needs and concerns of those affected and by helping people connect to information, services and social supports [15–17]. The three main principles of PFA are to look (for safety or for who needs help) listen (to those that are in distress) and link (to further support). Evidence exists to suggest that PFA is a useful intervention to reduce the initial effects of trauma [16], however findings are typically based on studies based in war or natural disasters such as earthquakes and floods [17, 18]. Recent studies suggest that PFA might be useful for reducing the initial distress caused by traumatic events and fostering resilience in healthcare professionals [16, 19]; however, systematic reviews [17, 18] have suggested that further research is needed to strengthen recommendations for more widespread use of PFA to support people following crises.

A recent scoping review [20] exploring applications of PFA across all populations concluded that PFA was beneficial in improving understanding of appropriate psychosocial responses to trauma and stress, and increasing self-efficacy by improving skills to support others in acute distress. However, the reviewers noted a lack of consistency in the reported outcomes associated with PFA within the included studies which limited their ability to synthesise findings, underscoring the need for clear reporting of this information in studies on PFA. Further, the review highlighted a need for clear guidance on how training should be delivered, the importance of ensuring that the intervention is tailored appropriately for the context in which it is being applied. The Wang et al. [20] review did not specifically consider the population of care home staff. Moreover, whilst a US study by Brown et al. [21] found that PFA training for nursing home staff improved the well-being of residents, no well-being outcomes for staff were reported in the study.

Historically, care home staff have been neglected in terms of research; however, given the evidence of the significant psychological impact of the pandemic on this population, there is a substantial need to understand the application and usefulness of well-being support tools, such as PFA, for this group.

### Objectives

This review therefore aimed to synthesise findings from studies exploring the usefulness of PFA for the well-being of nursing and residential care home staff.

## Method

The reporting of this review was informed by the PRISMA [22] guidelines for systematic reviews.

### Search strategy

A systematic search was conducted across 15 databases (Social Care Online, Kings Fund Library, Prospero, Dynamed, British Medical Journal Best Practice, Scottish Intercollegiate Guidelines Network (SIGN), The National Institute for Health and Care Excellence (NICE), Ovid, Proquest, Campbell Library, Clinical Trials, Web of Knowledge, Scopus, Ebsco CINAHL (Cumulative Index to Nursing and Allied Health Literature), and the Cochrane Library, to identify peer-reviewed articles published in English language from database inception to 20th June 2021. Search terms related to mental health first aid and psychological first aid, including psychological first aid (PFA), mental health first aid (MHFA), and nursing and care homes (nursing home, care home, residential home, residential care). Reference lists of relevant reviews were also screened [20]. The search strategy was designed and executed by a health specialist subject librarian.

### Eligibility

Full text, English language, peer-reviewed articles were eligible for inclusion if they presented original quantitative or qualitative research findings relating to well-being of nursing and residential care home staff who had received PFA training.

### Screening and results

**Fig. 1:**
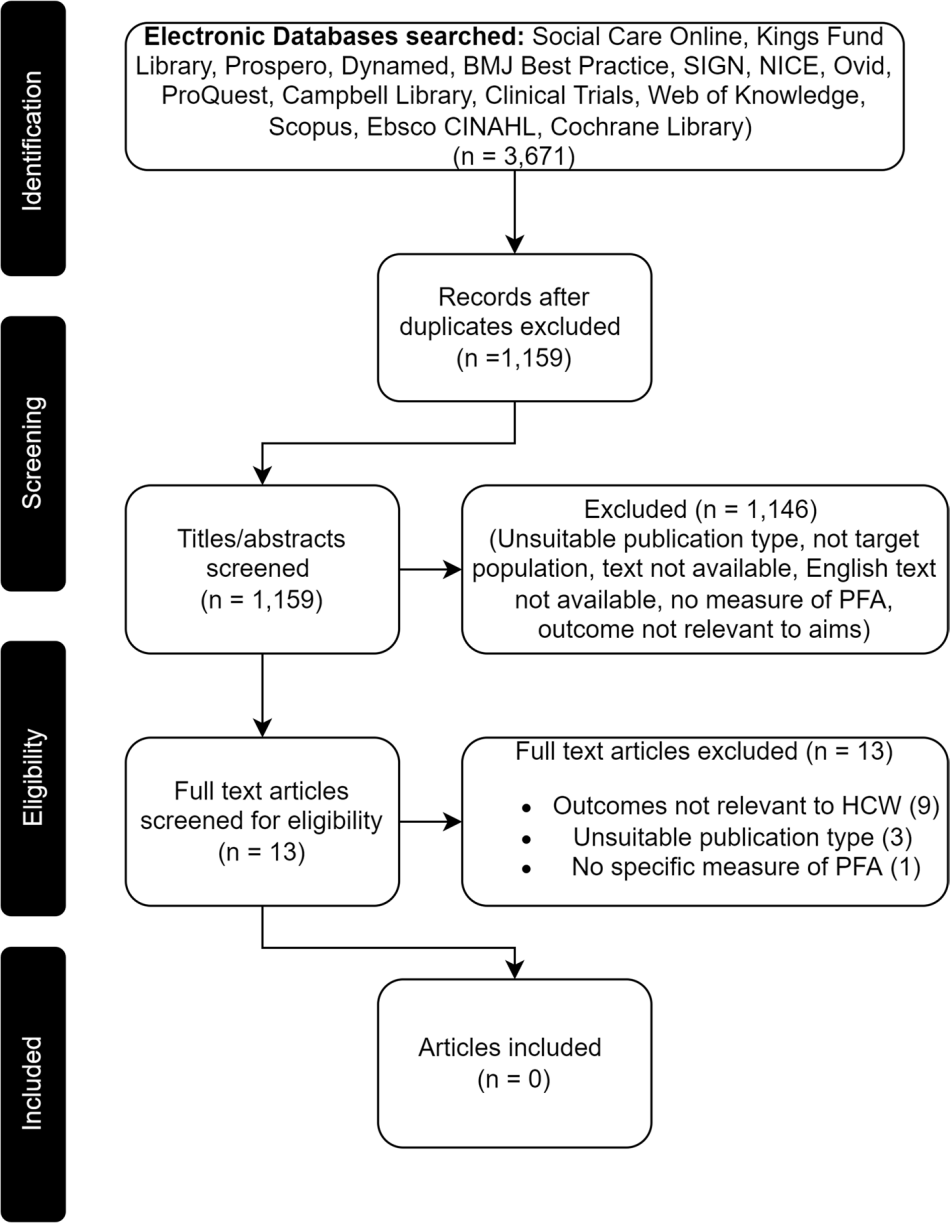
Adapted PRISMA Flow Diagram

Following removal of duplicates the screening list comprised 1,159 titles. At abstract and full text level, articles were independently screened by CM and MS, and reviewers met to discuss any discrepancies. Agreement percentages were calculated, and interrater reliability was quantified using Cohen’s kappa (*k*) (Fig. 1).

Of the 1,159 articles, 1,146 were excluded at title and abstract screening level (96%, *k* = 0.82). The remaining 13 articles were obtained at full text, all of which were then excluded (100%, *k* = 1). Figure 1 details this screening process.

## Discussion

The objective of this review was to systematically assess and synthesise the evidence from studies exploring the usefulness of PFA as a tool to support the well-being of nursing and residential care home staff. Unfortunately, no single study met our broad inclusion criteria. The results of this review were unexpected given that PFA, developed by the World Health Organisation [23] has been identified by Kisely et al. [9]; WHO 2020 [24, 25] and the COVID Trauma Response Working Group [19] as suitable for reducing initial distress caused by traumatic events among care workers. However, these findings align with earlier reviews that looked broadly at the use of PFA [18], victims of disaster or traumatic events [17] and first responders or volunteers in mass casualty events [26] that identified a lack of empirical evidence into the effectiveness of PFA training. However, the absence of empirical evidence for effective post disaster training or intervention, should not mean doing nothing. Doing nothing risks promoting a sense of lack of social support [17]. Lack of social support for people that have experienced disaster or traumatic events has been linked with the development of PTSD [27]. Thus, delivering a social, emotional, supportive, and pragmatic intervention that can reduce the initial distress and foster future adaptive functioning has the potential to protect against this. Hence, despite a paucity of evidence PFA is currently deemed as an acceptable intervention for those experiencing traumata [17] and for the last decade has been implemented by a number of organisations and government bodies responding to emergencies [28]. Unfortunately, even training in PFA has rarely been studied in care home settings with a recent review [20] identifying only one study [21] of implementation among 22 nursing home staff. What seems clear is that the initial promise of PFA recognised in other settings has not yet translated into care home settings. It is a priority to better understand how and why PFA might work and for whom in care home settings. Multiple adaptations and models of PFA have been implemented across settings and it is highly likely that care homes will require its own unique tailored implementation.

The reasons for the lack of empirical evidence could be manyfold, including practical, ethical, and contextual factors. For example, the burden to participants and the unpredictability of the timing of crisis events as well as responding in a timely manner could be a factor [29, 30]. Further, negative media attention surrounding the impact of COVID 19 in care homes may have resulted in staff being reluctant to engage in research during this time [31]. Challenges with recruitment pose a substantial barrier to conducting research with this population [32]. Another reason for the lack of empirical data could be attributed to the manual or domains that PFA covers. According to Hobfoll et al. [33], PFA covers five key aspects (a sense of safety, calming, a sense of self– and community efficacy, connectedness, and hope). While the main aim of PFA is to promote a sense of safety and a speeding return to comfort, the flexibility of PFA and how PFA is delivered, by whom, where and when in the disaster trajectory results in PFA interventions taking different forms make it more difficult to evaluate [34–36]. Similarly, no validated tool exists to assess the specific claimed outcomes associated with PFA. Lastly, as we know from behavioural sciences, there could be some reservations from some professionals towards evidence-based practice which can make it difficult to investigate the implementation of such an intervention [37]. This could be linked to a lack of awareness of healthcare staff about their own mental health needs or to the lack of awareness for the needs and resources for healthcare workers by organisations employing them or their governments [38]. Presumably, there is a still stigma attached to mental health in organisations which could cause hesitancy in help seeking among healthcare workers [39, 40]. However, findings relating to increases in difficulties with mental well-being within the care home workforce emphasise the need for effective interventions. The UK government recently launched an adapted PFA programme for those supporting the mental health of children and young people. This suggests scope for PFA to be adapted to meet the needs of different target populations. It is hypothesised that a PFA intervention tailored to the specific needs of care home staff would provide maximum benefit for this group.

## Strengths and limitations

One of the strengths of this systematic review is that a specialist health subject librarian developed the search strategy and searches. Secondly, we searched 15 separate databases which is very comprehensive. However, it is also important to recognise the limitations of this systematic review. Despite employing broad search terms and a sensitive search strategy there is no guarantee that we have not missed relevant literature that was not accurately indexed in the searched databases. We only reviewed papers in English language which is a limitation. Thirdly, we had rigorous selection criteria focusing on PFA interventions for HCWs and its effects on well-being, which might explain why no empirical studies could be included in the review. The lack of evidence for PFA interventions for HCWs does not prove evidence of the lack of usefulness of PFA, nor does it rule out the possibility that PFA may cause harm. Previous interventions, such as psychological debriefing, that were originally thought to be beneficial have been shown to be ineffective and potentially harmful [41]. This highlights the necessity for future studies focusing on the effectiveness of PFA for HCWs both in the context of the pandemic and beyond.

## Conclusion

This study highlights that the evidence of the impact of PFA on the well-being of nursing and residential care home staff is absent. PFA has likely been implemented as a recommendation to HCWs during the pandemic due to its face validity as a useful intervention for psychological support, despite the lack of evidence. The lack of evidence found here reinforces the urgent call to action to conduct methodologically robust studies which assess and cogently report the outcomes of PFA particularly in the under researched care home staff population.

## Data Availability

The datasets used and/or analysed during the current study are available from the corresponding author on reasonable request.

## Abbreviations

BMJ: British Medical Journal Best Practice
CINAHL: Cumulative Index to Nursing and Allied Health Literature
HCWs: Healthcare workers
MHFA: Mental health first aid MHFA
NICE: The National Institute for Health and Care Excellence
PFA: Psychological First Aid
PRISMA: Preferred reporting Items for Systematic Reviews and Meta-Analysis
PTSD: Post Traumatic Stress Disorder
SIGN: Scottish Intercollegiate Guidelines Network
USA: United States of America
